# Faecal incontinence as presentation of an ependymomas of the spinal cord

**DOI:** 10.1186/1477-7819-5-107

**Published:** 2007-09-25

**Authors:** Santhini Jeyarajah, Andrew King, Savvas Papagrigoriadis

**Affiliations:** 1 Colorectal and Histology Department, Kings College Hospital, Denmark Hill, London SE5 9RS, UK

## Abstract

**Background:**

Spinal tumours and ependymomas in particular are rare causes of cauda equina syndrome that present with faecal incontinence.

**Case presentation:**

We present a case of a 45 year old gentleman who presents to a colorectal clinic with incontinence. We then present a review of ependymomas with particular reference to the symptoms they cause as well a review of the neurophysiology of faecal continence.

**Conclusion:**

Suspicion for non-colonic causes for faecal incontinence should arise when there is absence of other etiologic factors. Establishment of the diagnosis of spinal tumours is with neurological examination and MRI spine.

## Background

Lower motor neuron symptoms such as lower limb weakness, loss of sensation, sphincter and erectile dysfunction can be caused by disruption of the cauda equina by a spinal tumour (cauda equine syndrome). The symptom of faecal incontinence due to primary spinal tumours however is rare. Is has been reported in lumbar schwannoma of the cauda equina, [1] lumbosacral intrapsinal lipomas [2,3] and metastatic spinal deposits [4]. Ependymomas however are not frequently associated with this symptom. Here we present a case where this is seen and explore the features usually associated with this condition.

## Case presentation

A 45 year old gentleman is referred to a colorectal clinic with diarrhoea and faecal incontinence for 2 months. He has also suffered with progressively worsening urinary symptoms: hesitancy, frequency and nocturia, poor stream and sensation of incomplete emptying and possible intermittent retention, with urge incontinence over 2 years. Previous review by Urologists revealed no abnormality on cystoscopy, X-ray KUB or renal ultrasound and a diagnosis of prostatitis was made. There was no history of pain, lower limb neurological symptoms and he had normal erectile function. There was no history of diabetes, multiple sclerosis or other neurological disease. On clinical examination, resting and squeeze anal tone were reduced. Perianal sensation was intact. Colonoscopy was requested which revealed a normal colon. Faecal calprotectin and stool cultures were normal. At this stage anorectal physiology was not requested as primary anorectal pathology was not thought to be the cause of these symptoms and there was a clear clinical picture of incontinence therefore manometry would not have altered management. A referral was made to Neurology and re-referral to Urologists.

Neurological review 1 month later revealed newly developed mild lower limb numbness when sitting down which disappeared on walking. However there was no deterioration in function. Full central and peripheral neurological examination only revealed a brisker reflex in the right knee.

**Figure 1:**
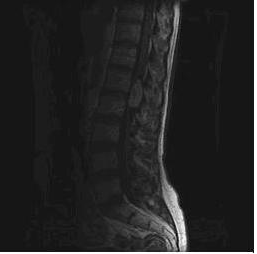
Ependymoma at level L1/L2 causing peripheral displacement of the cauda equina.

An MRI of the spine showed a lesion just distal to the conus, causing the roots of the cauda equina to be displaced peripherally at the level of L1/2. The lesion enhanced fairly uniformly and appearances were thought to be in keeping with an ependymoma (Figure 1).

**Figure 2:**
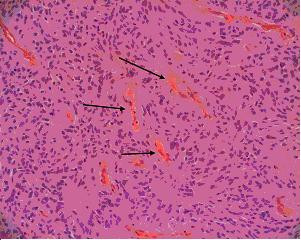
Histology from the ependymoma revealing relatively monomorphic cells with a somewhat fibrillary background and numerous perivascular pseudorosettes (arrows) (H&E).

A month later laminectomy and excision of this tumour was performed with electrophysiological intra-operative monitoring. Histology revealed a classical ependymoma (WHO Grade II) with no myxopapillary regions (Figure 2). Perivascular pseudorosettes were evident, and mitoses were very infrequent with no necrosis. The immunohistochemistry showed strong focal positivity for GFAP and S100, but only very focal positivity for EMA. The proliferation index was low at approximately 2%.

Six months after initial presentation he had some residual urinary symptoms but his faecal incontinence had resolved.

## Discussion

There are numerous causes of faecal incontinence ranging from anatomical (vaginal delivery, obstetric injury, anorectal surgery including lateral sphincterotomy, anal stretch, haemorrhoidectomy, surgery for fistula-in-ano, pelvic fracture, sphincter saving operations like low anterior resection, coloanal reservoir), congenital (anorectal malformations, cloacal defects, meningocoele, myelomeningocoele, imperforate anus, rectal agenesis), neurological (diabetes mellitus, multiple sclerosis, spinal cord lesions, pudendal neuropathy due to stretch nerve injury, central nervous system disorders such as stroke, trauma, infection, tumours) and a variety of functional causes [5]

### Anorectal anatomy & physiology

Faecal continence is regulated through integrated and coordinated activity between the anal sphincters and rectum. Reflex and voluntary activity of the internal and external sphincters, the puborectalis sling, rectal capacitance and sensitivity also contribute to this. Any abnormality in these factors can results in faecal incontinence, which affects about 1.4% of the population over 40 years of age in the United Kingdom [6]

The nervous supply mediating faecal continence is also complex. The anorectum and pelvic floor are supplied by sympathetic, parasympathetic and somatic fibers. Sympathetic preganglionic fibers originate from the lowest thoracic ganglion in the paravertebral sympathetic chain and join branches from the aortic plexus to form the superior hypogastric plexus. The hypogastric nerves unite with preganglionic parasympathetic fibers originating from ventral rami of the second, third, and often the fourth sacral nerves to form the inferior hypogastric plexus, located posterior to the urinary bladder. The inferior hypogastric plexus gives rise to the middle rectal plexus, vesical plexus, prostatic plexus, and uterovaginal plexus. The nerve supply to the rectum and anal canal is derived from the superior, middle, and inferior rectal plexus. Parasympathetic fibers in the superior and middle rectal plexuses synapse with postganglionic neurones in the myenteric plexus in the rectal wall. The pudendal nerve branches into inferior rectal, perineal, and posterior scrotal nerves. The inferior rectal nerve conveys motor fibers to the external anal sphincter, and sensory input from the lower anal canal as also the skin around the anus [7]

While cadaveric studies suggested the puborectalis was supplied by the pudendal nerve, electrophysiological stimulation studies in humans suggest this muscle is supplied, strictly ipsilaterally, by branches originating from the sacral plexus above the pelvic floor[8].

The anal canal itself is lined by numerous free and organized nerve endings (i.e. Meissner's corpuscles, Krause end-bulbs, Golgi-Mazzoni bodies and genital corpuscles). Sensory traffic is conveyed by unmyelinated small C fibres and larger A fibres. Anal relaxation induced by rectal distention is mediated by intrinsic nerves. This reflex is absent in Hirschsprung's disease. The extrinsic nerves are not essential for the reflex, as it is preserved in patients with cauda equina lesions or after spinal cord transaction [9]

The complexity of these mechanisms emphasise that defaecation is an integrated somato-visceral reflex and that the central nervous system plays a greater role in regulating anorectal sensorimotor functions compared with other regions of the gastrointestinal tract [10].

The elaborate somatic defecation response depends on centres above the lumbo-sacral cord, and probably craniad to the spinal cord itself.

### The role of ependymomas in faecal incontinence

Ependymomas are believed to account for 60% of all primary neoplasms of the spinal cord and filum terminale[11]. Most intraspinal ependymomas are considered to arise de novo from the region of the central canal, from the ventriculus terminalis of the conus, from within the filum terminale, or from cerebrospinal fluid (CSF) dissemination[12].

Intraspinal ependymomas are most easily grouped into 3 classes: intramedullary, myxopapillary ependymomas, and metastases from an intracranial origin. Intramedullary ependymomas commonly occur in the cervicothoracic part of the spinal cord [13,14] while myxopapillary lesions tend to occur in the conus and filum terminale[15]. Myxopapillary ependymomas tend to be WHO grade 1 and can be usually cured by resection when attached to the conus of filum terminiale. The intramedullary type are Grade II and therefore potentially more aggressive [16,17]. This case is unusual in that although it is at the level of the cauda equine it has the histological appearances of a classical intramedullary ependymoma rather than myxopapillary.

The most common symptom is pain in the back seen in 70–80% of patients, followed by sensory and motor neurological symptoms in 50–70% [18-20]. Although bladder dysfunction (13–21%) in the form of retention and over flow is more often a symptom [19-21], anal sphincter dysfunction is also associated with this condition but less commonly (15%) and tends to coexists with bladder dysfunction[21]. It however tends to present as constipation rather than diarrhoea and incontinence Sphincter dysfunction also appears to be a symptom that rarely occurs without coexistent pain and neurology as seen in this patient [18,19]. Sexual dysfunction occurs in up to 23% of patients [18].

Faecal incontinence being the primary presenting symptom in the absence of other neurological symptoms or back pain has not been reported.

MRI of the spine is the investigation of choice and complete surgical resection is the treatment for intraspinal ependymomas [13,14,19,22]. Total resection is generally curative, without the need for postoperative irradiation which is sometimes used when residual tumour remains depending on the level, type and grade of ependymoma.

## Conclusion

Spinal tumours and ependymomas in particular are very rare causes of faecal incontinence. Although diagnosis is easy when the patient presents with full cauda equina syndrome, it may be missed when the presentation is more atypical. Unexplained coexistence of faecal and urinary symptoms in a male should raise suspicion of a spinal lesion particularly in the absence of obstetric trauma, pelvic floor or anal surgery.

## Competing interests

The author(s) declare that they have no competing interests.

## Authors' contributions

The patient was first seen by SP in a clinical context who provided initial consultation and further management.

SJ was primary author, performed the review of medical records of the patient and a literature review for this article supervised by SP.

AK was the histopathologist who diagnosed the tumour.

All authors read and approved the final manuscript.
